# A Structural Comparison of Ordered and Non-Ordered Ion Doped Silicate Bioactive Glasses

**DOI:** 10.3390/ma13040992

**Published:** 2020-02-22

**Authors:** Seray Schmitz, Ana M. Beltrán, Mark Cresswell, Aldo R. Boccaccini

**Affiliations:** 1 Institute of Biomaterials, University of Erlangen-Nuremberg, Cauerstrasse 6, 91058 Erlangen, Germany; seray.kaya@fau.de; 2 Department of Materials and Transport Science Engineering, University of Seville, 41011 Seville, Spain; abeltran3@us.es; 3 Lucideon Ltd., Queens Road, Stoke-on-Trent ST4 7LQ, UK; Mark.Cresswell@lucideon.com

**Keywords:** mesoporous, ordered, non-ordered, bioactive glass, sol-gel method

## Abstract

One of the key benefits of sol-gel-derived glasses is the presence of a mesoporous structure and the resulting increase in surface area. This enhancement in textural properties has a significant effect on the physicochemical properties of the materials. In this context the aim of this study was to investigate how sol-gel synthesis parameters can influence the textural and structural properties of mesoporous silicate glasses. We report the synthesis and characterization of metal ion doped sol-gel derived glasses with different dopants in the presence or absence of a surfactant (Pluronic P123) used as structure-directing templating agent. Characterization was done by several methods. Using a structure directing agent led to larger surface areas and highly ordered mesoporous structures. The chemical structure of the non-ordered glasses was modified to a larger extent than the one of the ordered glasses due to increased incorporation of dopant ions into the glass network. The results will help to further understand how the properties of sol-gel glasses can be controlled by incorporation of metal dopants, in conjunction with control over the textural properties, and will be important to optimize the properties of sol-gel glasses for specific applications, e.g., drug delivery, bone regeneration, wound healing, and antibacterial materials.

## 1. Introduction

Mesoporous materials (MMs) are defined as materials having pores between 2 to 50 nm according to IUPAC (International Union of Pure and Applied Chemistry) [1]. They are used commonly in industry as catalysts, adsorbents, and for chromatographic applications [2,3]. Recently, the potential of mesoporous materials to be used for biomedical applications such as drug delivery systems and bone regeneration [4,5] has become more widely investigated. Further enhancement of the properties of mesoporous materials can be achieved by controlling the structural nature of their inherent porous network. A common method for doing this is to introduce structure-directing templating agents during the synthesis of mesoporous materials in order to generate products with highly ordered internal porous structures as opposed to porous structures that are non-ordered. Ordered MMs have important features such as long-range ordered porosity, narrow pore size distributions, and high surface areas. In addition, variable final mesostructures (e.g., rods, 3D structures, or sheets) can be obtained by modification of the type or amount of the surfactant acting as the structure directing mesophase template [6]. Surfactants can be divided into three groups; cationic, anionic, and non-ionic [7,8]. As a cationic surfactant, cetyltrimethylammonium bromide (CTAB) is mostly used. Also, Gemini surfactants, multihead group surfactants, and cationic fluorinated surfactants can be alternative templates to produce different mesoporous structures. These surfactants have specific properties such as excellent solubility, high critical micelle temperature, and they can be used in both acidic and basic media. On the other hand, they are toxic and relatively expensive [8]. Examples of anionic salt surfactants are carboxylates, sulfates, sulfonates, phosphates. In comparison to cationic surfactants, the repulsive interactions between anionic surfactants and silicate species do not lead to ordered mesostructures [8]. The mostly used non-ionic surfactants are oligomeric alkyl Poly(ethylene oxide) (PEO) surfactants, amphiphilic triblock–copolymers, sorbitan esters, etc. Non-ionic surfactants are non-toxic, biodegradable, and have relative low costs as compared with other surfactants [8]. Widely used non-ionic surfactants in mesoporous bioactive glass synthesis are P123 and F127. The first mesoporous bioactive glass was synthesized in 2004 by Yan et al. using the combination of P123, F127 and B50–6600 as non-ionic surfactants [9]. Due to its preferable properties in comparison to other surfactants, in this study a non-ionic surfactant, P123, was used.

The benefits of mesoporous structures include i) the possibility of drug loading and controlled release from the mesopores, and ii) the ability to achieve consistent and controlled degradation due to the ordered and uniform structures obtained using structure-directing agents. These properties are important for biomedical applications (e.g., drug delivery, bone regeneration, wound healing, and antibacterial materials) of the mesoporous bioactive glasses. Ideally due to their ordered pore structure, ordered mesoporous glasses could be more effective in their bioactive behavior and drug delivery capability compared to non-ordered glasses. However, if doped with suitable therapeutic elements in the right amount, the non-ordered glasses can also become attractive for antibacterial applications, wound healing, and bone regeneration. Indeed mesoporous bioactive glasses doped with metal elements can degrade homogeneously, thus the release of the therapeutic metal ions can be controlled. Therefore, control of the mesoporosity characteristics plays an important role regarding the mentioned medical applications of mesoporous bioactive glasses.

The preparation of mesoporous silica and mesoporous silicate glasses by sol-gel method has been extensively studied [4,5,6,7,9,10,11]. The impact of the variation of different sol-gel process parameters on the structure of mesoporous silica is well known. In particular, the use of structure-directing surfactants to generate mesoporous silica with highly ordered porous structures expanded the potential application of these materials significantly. The use of the sol-gel methodology to produce mesoporous silicate glasses represented a natural progression from pure silica and it enabled sol-gel derived glasses with compositional functionality to be produced, dependent on the nature of the additional oxides present. Amongst these, mesoporous silicate glasses with enhanced bioactivity [9], antibacterial [11], and angiogenesis [12] properties have been produced. In addition to the beneficial properties which result from the modification of the glass composition, the textural properties of mesoporous silicate glasses have also been found to be critical in enhancing their performance.

Mesoporous silicate glasses (MSGs) synthesized with surfactants have enhanced surface area and higher pore volume and in the context of biomedical applications have an increased ability to induce in vitro apatite mineralization in simulated body fluid (SBF), whilst also having excellent cytocompatibility [13,14,15,16].

The purpose of this study was to synthesize MSGs with and without the use of structure directing templates (P123) in combination with different dopants (biologically active ions) and to evaluate the potential impact of such parameters on the textural and structural properties of the resultant glasses. The dopants chosen were Ag_2_O, CuO, ZnO, and CeO_2_. These oxides were chosen for their proven antibacterial properties [17,18,19]. They also exhibit osteogenesis and angiogenesis [4,12] promoting properties, thus being very useful biologically active dopants for MSGs. Undoped and Ag_2_O, CuO, ZnO, and CeO_2_-doped MSGs with compositions of (76-x)SiO_2_−13CaO−11P_2_O_5_–x(Ag_2_O, CuO, ZnO, and CeO_2_) (x = 5 wt%) were synthesized by sol-gel (non-ordered MSGs) and surfactant-assisted sol-gel methods (ordered MSGs). A non-ionic surfactant was applied in the sol-gel process to synthesize the ordered MSGs.

## 2. Materials and Methods

### 2.1. Materials

Tetraethyl orthosilicate (TEOS, 98%, Sigma-Aldrich, Steinheim, Germany), triethyl phosphate (TEP, 99.8%, Sigma-Aldrich, Steinheim, Germany), calcium nitrate tetrahydrate (Ca(NO_3_)_2_·4H_2_O, 99%, Sigma-Aldrich, Steinheim, Germany), silver nitrate (AgNO_3_, 99.5%, Sigma-Aldrich, Steinheim, Germany), copper(II) nitrate hemi (pentahydrate) (Cu(NO_3_)_2_·2.5H_2_O, 99.99%, Sigma-Aldrich, Steinheim, Germany), zinc nitrate hexahydrate (Zn(NO_3_)_2_·6H_2_O, 98%, Sigma-Aldrich, Steinheim, Germany), cerium(III) nitrate hexahydrate (Ce(NO_3_)_3_·6H_2_O, 99.999%, Sigma-Aldrich, Steinheim, Germany), nitric acid (HNO_3_, 69%, Sigma-Aldrich, Steinheim, Germany), hydrochloric acid (HCl, 37%, Sigma-Aldrich, Steinheim, Germany), ethanol (C_2_H_5_OH, 96%, Sigma-Aldrich, Steinheim, Germany), Pluronic P123 (M_n_ 5800, Sigma-Aldrich, Steinheim, Germany), and distilled water were used without further purification for the preparation of non-ordered and ordered MSGs.

### 2.2. Method

#### 2.2.1. Sol–gel Synthesis of Non-ordered mesoporous silicate glasses (MSGs)

Silica-based undoped and Ag_2_O, CuO, ZnO, and CeO_2_-doped MSG compositions [76SiO_2_−13CaO−11P_2_O_5_–5(Ag_2_O, CuO, ZnO, and CeO_2_)] were prepared by the sol–gel method. In the first step of undoped-MSG preparation, 50 mL TEOS was mixed with 33.12 mL of distilled water. As a catalyst 6.80 mL 0.5 M HCl was also added and mixed for 30 min. The preparation of doped-MSGs required 46.80 mL of TEOS. The catalyst used for Ag, Zn, and Ce-MSGs was 24.04 mL of 0.5 M HNO_3_ and for Cu-MSGs 17.50 mL of 0.5 M HCl. In the next step, 4.76 mL TEP was added to the solution and 30 min mixing was done. Afterwards, 9.87 g of Ca(NO_3_)_2_·4H_2_O was added to the solution and stirred for 60 min. For the doped-MSGs, the nitrates of the dopants were added to the sol and stirred for a further of 60 min. The resultant sols were aged for 3 days under a fume hood without stirring, and the gels were dried at 40 °C for 48 h. In the last step, to obtain undoped, Cu, Zn, and Ce-MSGs, heat treatment at 700 °C with 233.33 °C/h heating rate for 3 h was applied. For the Ag-MSGs, the heat treatment was carried out at 500 °C with a 166.66 °C/h heating rate for 5 h. Afterwards, the glasses were left for slow cooling in the furnace.

#### 2.2.2. Surfactant-Assisted Sol-gel Synthesis of Ordered MSGs

MSGs with ordered mesoporosity were synthesized by evaporation induced self-assembly (EISA) [20] process with a non-ionic surfactant Pluronic P123. 4 g of P123 was stirred into 76 mL of 96 vol% ethanol and 1.00 mL of 0.5 M HCl (for undoped and Cu-MSGs) for at least 1 h until it became a clear solution. 1 mL of 0.5 M HNO_3_ was used as the catalyst for Ag, Zn, and Ce-MSGs. After P123 dissolution, 7.13 mL TEOS was added to the solution and stirred for 30 min for undoped-MSGs. Doped-MSGs required 6.65 mL of TEOS. In the next step, 0.67 mL of TEP was added and stirred for a further 30 min. A total of 1.40 g of Ca(NO_3_)_2_·4H_2_O was added and stirred for 1 day for undoped-MSGs. For the doped-MSGs, after stirring 1.40 g of Ca(NO_3_)_2_·4H_2_O for 1 h, the nitrates of the dopants were added to the sol and stirred for 1 day. In the next step, these sols went through evaporation induced self-assembly (EISA) process, by leaving the sols in petri dishes under a fume hood. Finally, heat treatment was done at 700 °C at a 233.33 °C/h heating rate for 3 h in the case of undoped, Cu-MSG, Zn-MSG, and Ce-MSG synthesis. Ag-MSGs were obtained at 500 °C at a 166.66 °C/h heating rate for 5 h. Afterwards, the glasses were left for slow cooling in the furnace.

### 2.3. Characterization Methods

#### 2.3.1. High Resolution Transmission Electron Microscopy (HRTEM)

High resolution transmission electron microscopy (HRTEM) studies were performed with a FEI Talos F200S microscope (ThermoFisher Scientific, Eindhoven, Netherlands) equipped with Super-X energy dispersive X-ray spectrometry (EDX) system, including two silicon drift detectors. The microscope operates at an accelerating voltage of 200 kV. Prior to the HRTEM analyses, MSG powders were deposited on a Holey carbon film on a copper grid. The pore profiles were obtained by using ImageJ analysis to observe the distance between the pore channels. Twenty-five to 150 pore size measurements were done on the MSG samples. The standard deviations of these measurements are also given.

#### 2.3.2. Nitrogen Adsorption Analysis (Brunauer-Emmett-Teller (BET) and Barrett-Joyner-Halenda (BJH))

The mesoporous properties (surface area, pore size diameter, and pore volume) of the undoped and doped-MSGs were investigated by nitrogen adsorption porosimetry. The machine used was TriStar II Plus 2.03 (Micromeritics, Norcross, GA, USA). Before performing the adsorption analyses, MSGs were degassed under vacuum for 5 h at 423 K. The analyses were performed at 77 K. Brunauer-Emmett-Teller (BET) method was used to determine the surface area of the MSGs. Barrett-Joyner-Halenda (BJH) method was applied to determine the pore size diameter of the MSGs from the desorption branch of the isotherm.

#### 2.3.3. Fourier Transform Infrared Spectroscopy (FTIR)

Undoped and doped-MSGs were placed directly onto the diamond crystal (spectral range: 4000–400 cm^−1^) of a GladiATR Vision (PIKE Technologies Ltd., Madison, Wisconsin, USA), attenuated total reflectance accessory that was installed in the sample compartment of an Agilent 660-IR FTIR spectrometer (Varian Australia Pty Ltd., Melbourne, Victoria, Australia). A press was used to secure the samples in place and to ensure that a sufficient homogeneous contact between the sample and attenuated total reflectance (ATR) crystal was achieved. The samples were measured in the region 4000–400 cm^−1^ wavenumbers and 32 scans were collected at a resolution of 4 cm^−1^.

#### 2.3.4. Semi-quantitative X-ray Fluorescence (XRF)

To determine the elemental wt% compositions of the MSGs, semi-quantitative X-ray Fluorescence (XRF) method was applied. Axios wavelength dispersive (WD) XRF spectrometer (Panalytical, Almelo, Netherlands) was used.

#### 2.3.5. Powder X-ray Diffraction (XRD)

The X-ray diffraction (XRD) analyses of the MSGs were done by D8 Advance (Bruker, Karlsruhe, Germany), with Copper Kα radiation at 40 kV and 30 mA. The diffraction range of the analyses was 5 to 70° 2θ. The step size and the dwell time used to collect the data using Bragg-Brentano geometry was 0.02° 2θ and 0.5 s per step, respectively. X-rays were detected simultaneously by a Lynxeye solid state detector over a 3° 2θ window.

## 3. Results

### 3.1. High Resolution Transmission Electron Microscopy (HRTEM)

**Figure 1:**
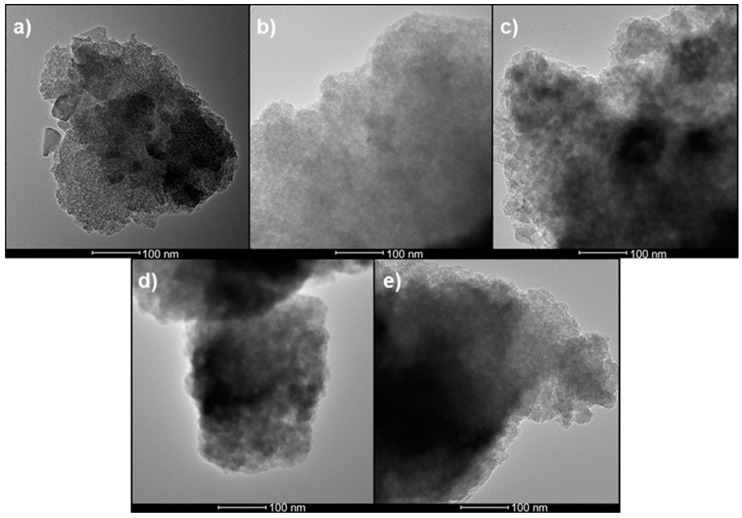
High resolution transmission electron microscopy (HRTEM) images of non-ordered mesoporous silicate glasses (MSGs): (**a**) Undoped-MSG; (**b**) 5 wt% Ag-MSG; (**c**) 5 wt% Cu-MSG; (**d**) 5 wt% Zn-MSG; (**e**) 5 wt% Ce-MSG. Scale bar = 100 nm.

Figure 1 shows HRTEM images of the undoped and 5 wt% metal-doped non-ordered mesoporous glasses. It can be seen that the MSG particle surfaces have a globular and textured nature which is typical for materials synthesized by sol-gel method. These MSGs did not show any ordered mesoporous network.

**Figure 2:**
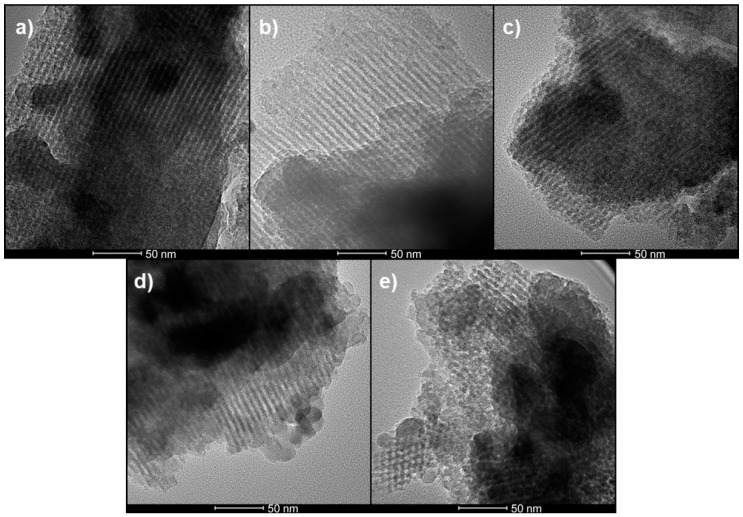
HRTEM images of ordered MSGs: (**a**) Undoped-MSG; (**b**) 5 wt% Ag-MSG; (**c**) 5 wt% Cu-MSG; (**d**) 5 wt% Zn-MSG; (**e**) 5 wt% Ce-MSG. Scale bar = 50 nm.

In contrast, Figure 2 shows the highly ordered porous networks associated with the ordered undoped and 5 wt% metal-doped mesoporous glasses. Except 5 wt% Ce-MSG (Figure 2e), in the images of other ordered MSGs, just the lateral view (along the length of mesopores) of the mesoporous structures were seen. In the case of 5 wt% Ce-MSG (Figure 2e), in addition to the lateral view of pore channels, a face-on view of a honeycomb structure was seen. ImageJ was used to measure the average pore size diameters of the ordered MSGs.

**Figure 3:**
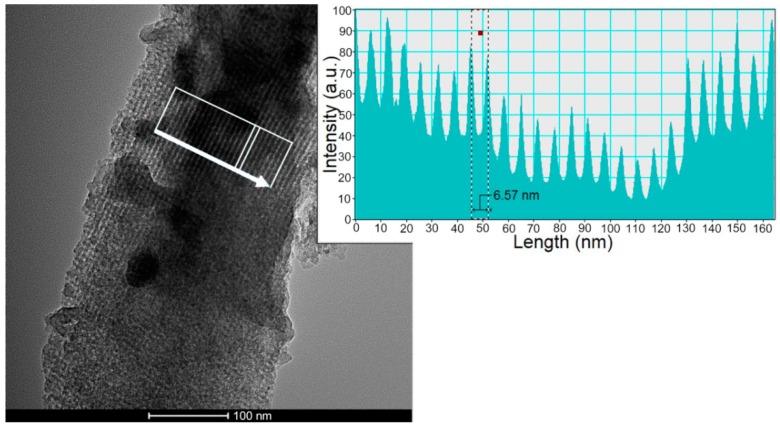
Pore size determination of an ordered MSG.

Figure 3 shows an example of how the average pore size was typically determined; the arrow indicates the direction along which the profile has been plotted. In order to increase the intensity, the profile has been integrated over the area. The measurements were taken from the well-ordered lateral areas of the MSGs. Ordered undoped-MSGs have a pore size diameter of 6.67 ± 0.33 nm. Ordered 5 wt% Ag-MSGs have pore sizes of 6.66 ± 0.20 nm. The pore sizes of ordered 5 wt% Cu-MSGs, 5 wt% Zn-MSGs, and 5 wt% Ce-MSGs are 6.22 ± 0.63 nm, 6.58 ± 0.92 nm, and 6.34 ± 0.15 nm, respectively.

The average pore diameters of the ordered MSGs are shown in Table 1.

### 3.2. Nitrogen Adsorption—BET and BJH

#### 3.2.1. Non-ordered and Ordered Undoped Mesoporous Glasses

**Figure 4:**
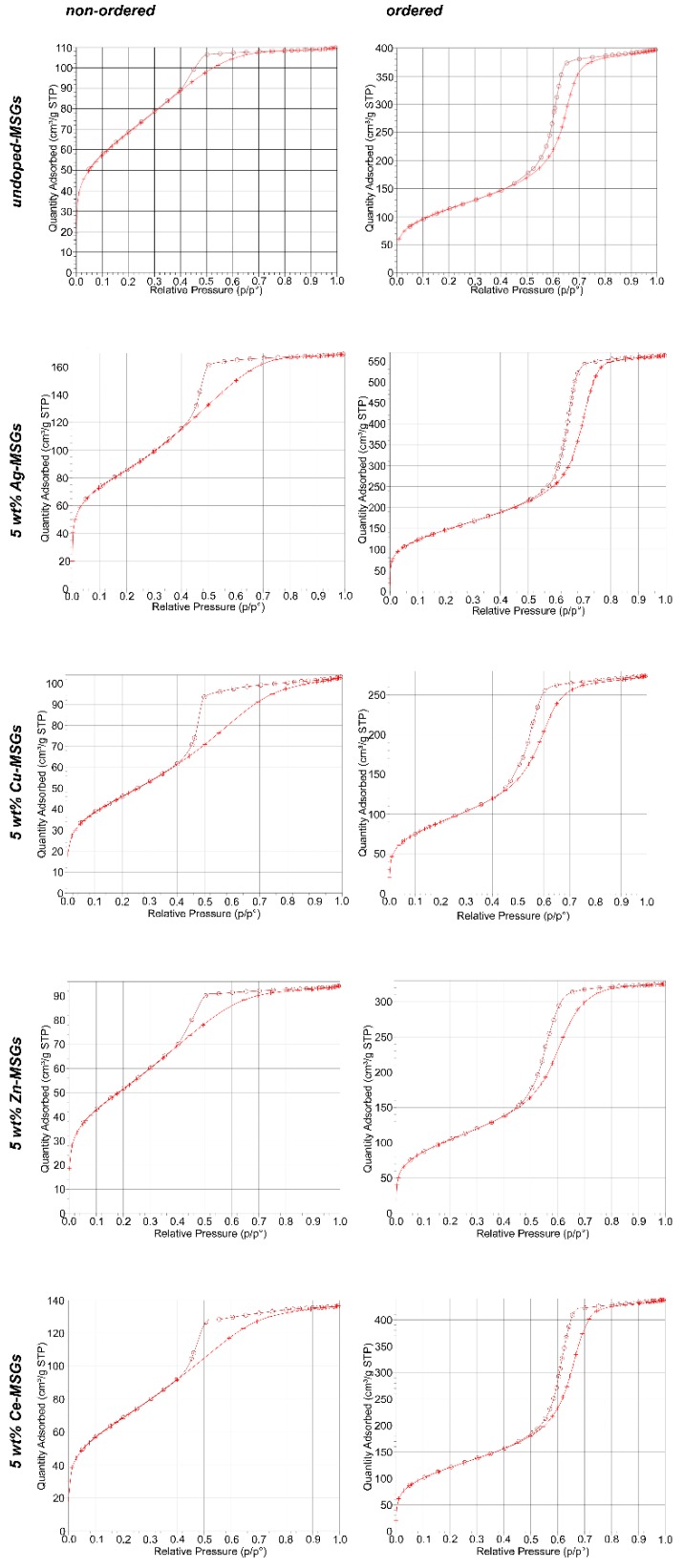
N_2_ adsorption-desorption isotherms of non-ordered and ordered, undoped-MSGs, 5 wt% Ag-MSGs, 5 wt% Cu-MSGs, 5 wt% Zn-MSGs, and 5 wt% Ce-MSGs. Crosses (+) and open circles (O) represent the adsorption and desorption branches, respectively.

According to the N_2_ adsorption-desorption isotherms of non-ordered and ordered, undoped-MSGs, both the non-ordered and ordered undoped-MSGs show type IV isotherms (Figure 4), which is characteristic of mesoporous materials [11,21]. The hysteresis type of non-ordered undoped-MSG is H2 (Figure 4), which is associated with ink-bottle shape pores, whilst the hysteresis type of ordered undoped-MSG is H1 (Figure 4), which is characteristic of cylindrical pores. H1 hysteresis loop of the ordered undoped-MSG has parallel adsorption and desorption branches that are almost vertical at the capillary condensation region. In comparison H2 hysteresis loop of non-ordered undoped-MSG has a steeper desorption branch than its adsorption branch. The pore properties of these MSGs are given in Table 2.

#### 3.2.2. Non-ordered and Ordered 5 wt% Ag-, Cu-, Zn, and Ce-doped Mesoporous Glasses

The hysteresis types of non-ordered 5 wt% Ag-, Cu-, Zn-, and Ce-MSGs are H2 (Figure 4), while the hysteresis types of ordered 5 wt% Ag-, Cu-, Zn-, and Ce-MSGs are H1 (Figure 4). The hysteresis curves of ordered 5 wt% Ag-, Cu-, Zn-, and Ce-MSGs have parallel adsorption and desorption branches that are almost vertical at the capillary condensation region, which is characteristic of cylindrical pores. In contrast, the H2 hysteresis of non-ordered 5 wt% Ag-, Cu-, Zn-, and Ce-MSGs have steeper desorption branches than their adsorption branches. The values of the porosity-related properties (surface area, pore volume, and pore size diameter) of ordered 5 wt% Ag-, Cu-, Zn-, and Ce-MSGs are higher than those of the non-ordered 5 wt% Ag-, Cu-, Zn-, and Ce-MSGs. The relevant properties of these MSGs are given in Table 2.

### 3.3. Fourier Transform Infrared Spectroscopy (FTIR)

**Figure 5:**
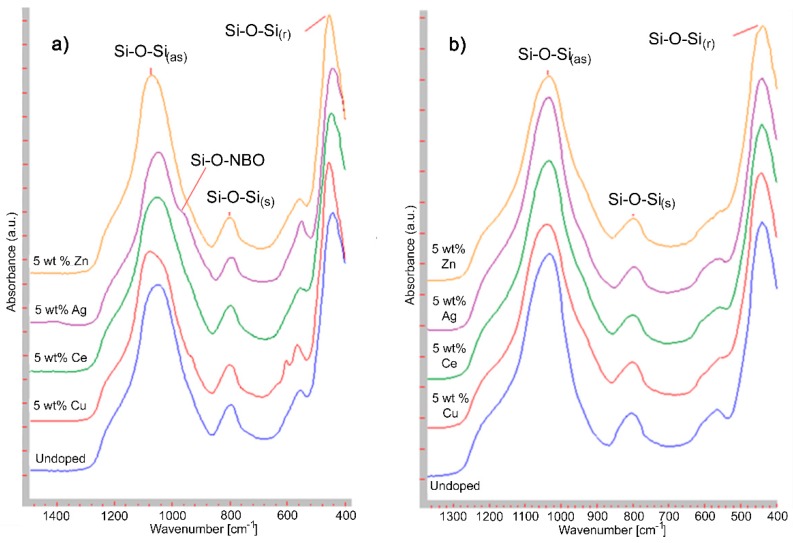
Fourier transform infrared spectroscopy (FTIR) spectra of: (**a**) Non-ordered mesoporous glasses; (**b**) ordered mesoporous glasses. (r) Shows rocking, (as) is asymmetric and (s) is symmetric.

The portion of the vibrational spectra of most interest (~1400–400 cm^−1^) for the non-ordered mesoporous glasses can be seen in Figure 5a and for the ordered mesoporous glasses in Figure 5b. This region is of primary importance since it is dominated by the vibrational bands associated with Si-O. All samples show strong and broad bands in the range 1300–1000 cm^−1^, which can be assigned to the asymmetric Si-O-Si stretching mode [22]. Absorption bands centered at ~800 cm^−1^ in all samples result from symmetric Si-O-Si stretching modes [23], whilst bands at ~450 cm^−1^ can be attributed to rocking vibration modes of Si-O-Si [22,24]. Small shoulders of the main Si-O-Si stretching band at ~950 cm^−1^ are only visible for the non-ordered mesoporous Ag and Cu- MSGs and can be assigned as Si-O non-bonding oxygens (NBO) [22,23]. Absorption bands at ~560 cm^−1^ can be assigned as bending P-O vibrational mode [22,25]. In addition, all materials show a broad band (shown in the supplementary section (Figures S1 and S2) as an example for ordered 5 wt% Cu-MSG) at 3700–3000 cm^−1^ attributable to various vibrational modes of hydroxyl groups; hydrogen-bonded and non-hydrogen-bonded silanols and physisorbed water [22].

The main vibrational bands and associated approximate peak wavenumbers can be seen in Table 3.

### 3.4. Semi-quantitative X-ray Fluorescence

The non-ordered and ordered, undoped, and 5 wt% doped mesoporous glasses were examined for their experimental wt% compositions by semi-quantitative (SQ) X-ray fluorescence technique. Results are shown in Table 4 and Table 5.

Even if the XRF results could be prone to variations, the data showed that the reproducibility of the compositions of the non-ordered MSGs was more consistent and their experimental compositions were closer to their theoretical values compared to ordered MSGs. Among the different dopant (Ag, Cu, Zn, and Ce) incorporated ordered MSGs, there were large compositional variabilities. Based on their theoretical 5 wt% dopant values, non-ordered 5 wt% Ag-, Cu-, Zn-, and Ce-MSGs had higher dopant incorporation than the same composition ordered MSGs as shown in Table 4.

### 3.5. Powder X-ray Diffraction (XRD)

**Figure 6:**
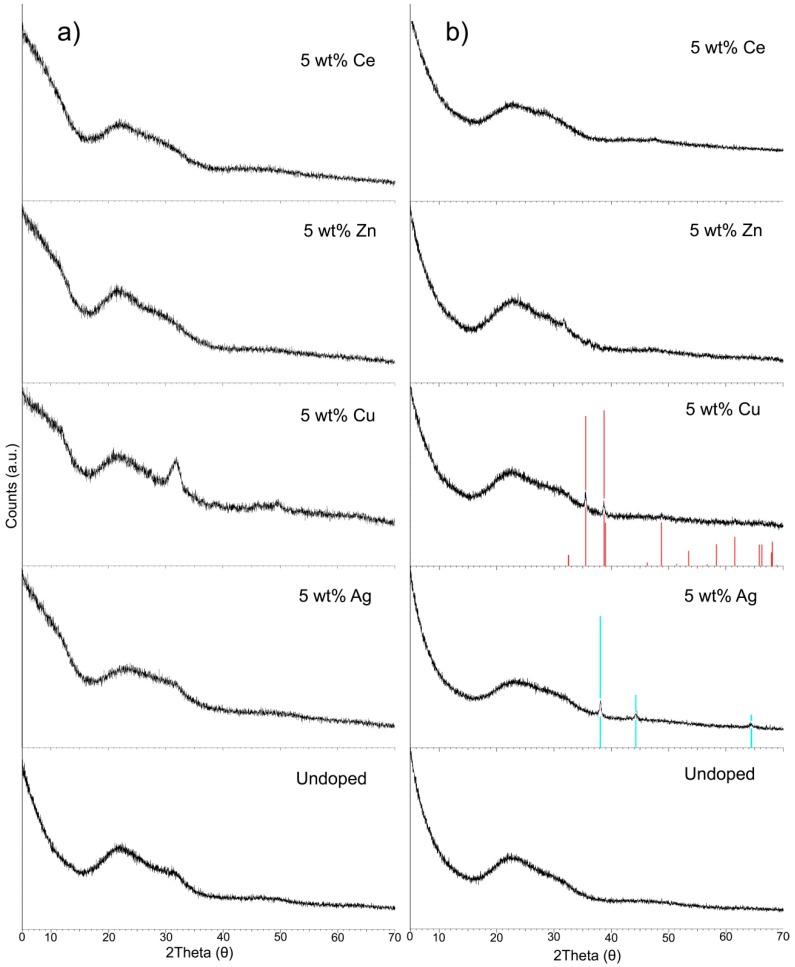
X-ray diffraction (XRD) spectra of: (**a**) Undoped and doped non-ordered mesoporous glasses; (**b**) undoped and doped ordered mesoporous glasses.

All of the materials studied exhibit the broad amorphous halo between 15° and 40° 2θ characteristic of amorphous silicate glasses (Figure 6). Of the non-ordered mesoporous glasses, only the Cu-doped glass contains any additional diffraction peaks. As shown in Figure 6a, there is a broad peak which is centered at ~30–32° 2θ. The peak fitting software and XRD analysis database could not identify this peak. Additional diffraction peaks were also seen in the ordered mesoporous glass systems. Face centered-cubic silver (ICDD PDF Card No. 04-0783 Ag Silver-3C, syn) and copper oxide peaks (ICDD PDF Card No. 72-0629, CuO Tenorite) were identified in the Ag-doped ordered MSG and Cu-doped ordered MSG, respectively (Figure 6b).

## 4. Discussion

As shown by the HRTEM images (Figure 1 and Figure 2), the non-ordered and ordered mesoporous structures of undoped and 5 wt% metal-doped (Ag, Cu, Zn, and Ce) MSGs can be compared. When no structure-directing templating surfactant was present during the sol-gel process, the product MSGs had non-ordered mesoporous structures (Figure 1). The addition of P123 surfactant to the sol-gel process, which acts as a template to generate an ordered mesoporous structure, led to ordered 2D hexagonal mesoporous structures, as shown in Figure 2. As revealed by ImageJ analysis of the HRTEM micrographs of the ordered MSGs, there were no significant differences in the average pore diameters between the materials irrespective of the nature of the dopant. It can be suggested that the dopant type is not a dominant factor on the pore sizes of the ordered MSGs obtained by HRTEM.

The relative minor trends in pore size diameters of the ordered 5 wt% Cu and 5 wt% Zn-MSGs compared to ordered undoped-MSG, as identified from the HRTEM micrographs, are corroborated following suitable interpretation of the N_2_ adsorption isotherms. Pore size diameter of the ordered undoped-MSG was seen to decrease with the incorporation of CuO and ZnO (Table 2). A similar case was also seen in a previous study in which the pore size diameter of Cu-doped BGs was lower than its undoped version [26]. ZnO addition also results in lower pore sizes of ordered MSGs [27]. Although ordered 5 wt% Ag-MSG did not exhibit any difference in its HRTEM measured pore size compared to ordered undoped-MSG (Table 1), low amounts of Ag_2_O (Table 4) present in undoped-MSG increased the BJH measured pore size diameter (Table 2). This situation was observed in a study, in which 80SiO_2_-15CaO-5P_2_O_5_ (mol%) MBG was doped with 0.8 mol% Ag_2_O and the resultant Ag-MBG had larger pore size compared to its undoped equivalent—7 nm [11] vs. 5 nm [28]. Ordered 5 wt% Ce-MSG shows a decreasing trend of HRTEM measured pore size compared to ordered undoped-MSG (Table 1), however it exhibits a higher BJH measured pore size (Table 2).

In contrast, the nitrogen adsorption data show that the pore diameters of the non-ordered MSGs all increased with the addition of the dopant metal oxides relative to the undoped-MSG. This is an important result that suggests that in the absence of a structure-directing template, the nature of the dopant could have more significant impact on the non-ordered MSGs’ textural properties. For the non-ordered glasses, Ag, Cu, and Ce were found to be quantitatively incorporated into the MSG structure, whilst only 75% of the intended Zn content was found to be present, as seen from the XRF mol% composition data (Table 5). CuO incorporation increased the pore size the most (Table 2), followed by Ce, Ag, and the least change was seen in the Zn-MSG, possibly attributable to its lower incorporation into the glass network. It has to be noted that the pore size diameters of the ordered MSGs as determined by nitrogen adsorption analysis were lower than the pore sizes as measured from the HRTEM images. Recent studies performed on materials which have mesopore diameters less than 10 nm have shown that the mesopore size can be underestimated when the BJH model (modified Kelvin equation) is used [29]. This situation is also observed in the MSGs in this study.

In all cases, the ordered MSGs had increased textural properties relative to the equivalent non-ordered MSGs. The ordered MSGs had BET surface areas in the range 1.7–2.0 times higher than the non-ordered MSGs, whilst the pore volumes of the ordered MSGs were in the range 2.7–3.7 times higher than the equivalent non-ordered MSGs (Table 2).

The types of hysteresis loops of all of the non-ordered MSGs were H2 type (Figure 4), which is characteristic of narrow mouth (ink-bottle shape) and relatively uniform channel-like pores [9,30]. The types of hysteresis loops of ordered MSGs were H1 type (Figure 4). This hysteresis type is associated with cylindrical pores, attributed to highly ordered mesoporous materials [31].

A more disrupted network is formed when the network modifier oxides are incorporated into a glass network. The glass network connectivity decreases as a result of the local defects formed as bridging oxygens are replaced by non-bridging oxygen (NBO) bonds [32]. This disruption of the glass network is a key factor for increasing the textural properties of the MSGs doped with therapeutic ions [11,33]. However, in the current study this effect was only noted for the incorporation of low molar amounts of metal dopant ions. For the non-ordered Cu and Zn doped-MSGs, where the mol% level of dopants incorporated into the structure was higher compared to other non-ordered MSGs, there was a decrease in the surface area as the mesoporous network was progressively destroyed [26]. With the addition of Ag_2_O into non-ordered undoped-MSG, the surface area increased (Table 2), in line with similar results observed previously [11]. Moreover, incorporation of CeO_2_ into non-ordered undoped-MSG network also resulted in a slight increase in the surface area.

Likewise, similar changes in surface area were seen for the ordered MSGs. Cu and Zn addition into undoped-MSG decreased the surface area (Table 2). In particular, Cu lowered the surface area the most due to the presence of a distinct CuO phase, as detected during the XRD analysis (Figure 6b). The presence of crystalline CuO has been shown to destroy the mesoporous structure leading to decreased surface area and pore volume [27]. As with the non-ordered MSGs, the addition of Ag and Ce to ordered MSGs resulted in increased surface areas (Table 2). A similar effect was also seen in a previous study, where 0.8 mol% Ag_2_O-doped 80SiO_2_-15CaO-5P_2_O_5_ MSG [11] had 528 m^2^/g surface area compared to 351 m^2^/g of undoped-MSG [11,28]. In most studies, cerium incorporation into MBGs was found to decrease the surface area [34,35]. In contrast to these, the results from the present study have shown that the surface area of Ce-MSG was increased. In order to better understand the changes in the pore properties, more detailed structural analyses would be needed (for instance, solid state NMR analysis, computer simulation, and small angle x-ray scattering experiments).

Only the Ag and Cu-doped non-ordered MSGs showed the Si-O-NBO stretch in their FTIR spectra (Table 3) [22,23]. This can be attributed to the successful incorporation of Ag and Cu into the MSG network to result in an increase in the number of Si-O-NBO bonds. The molar % XRF data also confirmed that Ag and Cu were fully incorporated into the glass structure (Table 5). All other peak band wavenumbers for the undoped and doped non-ordered MSGs only display relatively minor shifts, with the exception of the asymmetric Si-O-Si stretching band of the 5 wt% Cu and Zn-doped MSGs. These two bands shifted up field by ~24–30 wavenumbers relative to the undoped-MSG, possibly indicating a broad depolymerization of the glass network (breakage of Si–O–Si bridges, causing an increase in the number of Si–O– non-bridging terminal bonds) [36] of these two materials. Another reason for the higher wavenumber shift of the Si–O–Si asymmetric stretching vibration band could be due to the Si-O-Si bond angle change. The incorporation of relatively small metallic ions in the glass structure would result in a smaller Si–O–Si bond angle. This smaller angle leads to absorption at higher wavenumbers and therefore a shift of Si–O–Si asymmetric stretching band [27]. All of the MSGs showed PO_4_^3−^ bending vibration bands around 500–600 cm^−1^. In addition to these, for non-ordered 5 wt% Cu-MSG, at 604 cm^−1^ wavelength another PO_4_^3−^ bending vibration peak [22] was observed, which could be due to the more crystal-like arrangement of the phosphate groups.

Compared to non-ordered MSGs, the formation of Si-O-NBO bonds in the ordered MSGs was not clearly evident. This can be related to the lower level of dopant incorporation into the glass network as confirmed by XRF analysis (Table 4 and Table 5). In general, there was very little variation in the peak wavenumbers of all silicate vibrational bands across all samples as the dopant was varied. Again, this was due to the lower mol% of dopant present in the ordered MSGs.

It was seen from the semi-quantitative XRF results, that the dopant ions were not fully incorporated into the structures of ordered MSGs (Table 4 and Table 5). Non-ionic surfactant was used for the synthesis of ordered MSGs. When non-ionic surfactants are used as the template for ordered structure formation, a hybrid interface between the hydrophilic parts of the surfactant and the silanol groups (present on hydrolyzed silica species) [37] and dopant ions is formed. As the surfactant and nitrate removal occurs during calcination of the ordered-MSGs [37], the metallic ions which could not be incorporated in the MSG structure are remaining as elemental phases or oxides outside of the MSGs.

In comparison to ordered MSGs, non-ordered MSGs had better dopant incorporation (Table 4 and Table 5). The reason might be due to the absence of surfactant in the formation process of non-ordered MSGs, non-ordered structures of MSGs might have a higher capacity of ion incorporation.

**Figure 7:**
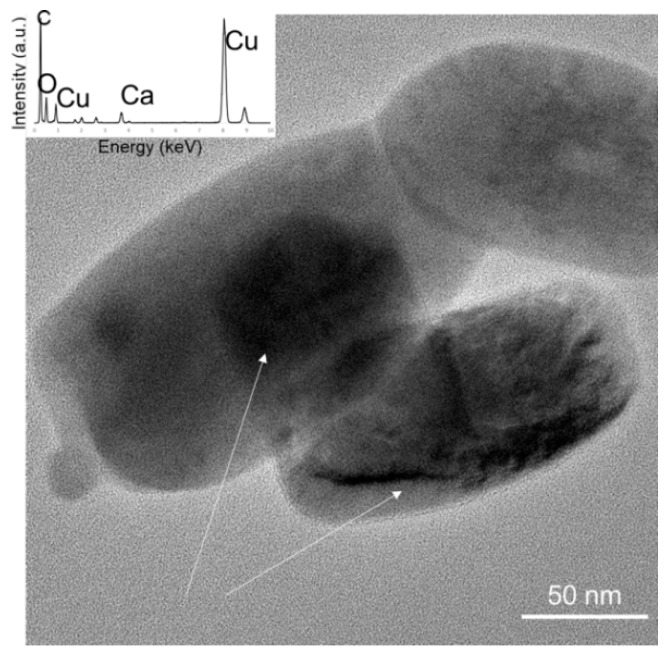
Cu included nanoparticles in HRTEM. Inset energy dispersive X-ray (EDX) profile acquired on the particle.

All non-ordered MSGs exhibit amorphous behavior with a halo between 15° and 40° 2θ characteristic of amorphous silicate glasses, as seen in Figure 6a. Only non-ordered 5 wt% Cu-doped MSG had an additional peak with a broad peak centered at ~30–32° 2θ. This additional peak could be due to the Cu included nanocrystalline particles, which are present in non-ordered 5 wt% Cu-doped MSG. The HRTEM analysis shows the presence of these nanoparticles (Figure 7). These particles could consist of either Cu or CuO. EDX analysis confirmed the presence of Cu in these particles (inset Figure 7). Oxygen was also detected in the spectra, but this might belong to the oxygen in the MSG composition.

Among the ordered MSGs, 5 wt% Ag and Cu-MSGs showed silver and copper oxide peaks in their XRD diffractograms (Figure 6b); 5 wt% Zn and Ce-MSGs were amorphous. Smaller deviations in some XRD spectra are not in a relevant level to affect the amorphous situation of the MSGs. Ag_2_O in the glass structure can reduce to Ag in the presence of UV light. It has been shown that the addition of silver in bioactive glasses can cause an increase of crystallization of quartz and metallic silver [38]. In ethanol environment, Ag^+^ ions have been shown to reduce to Ag [39]. In the synthesis of ordered Ag-MSGs in this study, ethanol was also used, which might have increased the extent of Ag^+^ reduction into metallic silver. Similarly, Cu^2+^ ions present in the sol were not completely incorporated into the gel network (as given by XRF data, Table 4 and Table 5), oxidation occurred during calcination and formed a CuO phase. The CuO phase was also observed in the XRD patterns of 5, 10, and 15 mol% CuO incorporated 80SiO_2_-15CaO-5P_2_O_5_ BG synthesized via P123 assisted sol-gel process [27]. It was seen that with increasing Cu content in the BG, the intensity of the CuO peaks were increased.

## 5. Conclusions

MSGs were synthesized by sol-gel method with and without a surfactant to obtain ordered and non-ordered mesoporosity, respectively. These MSGs were compared in terms of their structural properties. MSGs with highly ordered mesoporosity were obtained by the surfactant-assisted synthesis route and these MSGs exhibited larger surface areas and higher pore volumes than the non-surfactant assisted MSGs. FTIR spectra showed that the addition of dopant ions to non-ordered MSGs modified the glass network more than was the case with ordered MSGs due to a higher extent of dopant incorporation into the product glass structures. This was further demonstrated by XRF analysis. The addition of copper to both non-ordered and ordered MSGs was shown to decrease the surface area the most and it changed the position of the Si-O-Si vibration peaks in FTIR. The developed MSGs should be characterized further for example using solid state NMR, computer simulation, X-ray scattering experiments, Raman spectroscopy or XPS analysis and, in particular, such advanced characterization may help to explain the observed increase in textural properties for Ce-MSGs.

## Figures and Tables

**Table 1:** Average pore diameters of ordered MSGs.

Dopant	Pore Diameter (nm) ± Standard Deviation
—	6.67 ± 0.33
5 wt% Ag	6.66 ± 0.20
5 wt% Cu	6.22 ± 0.63
5 wt% Zn	6.58 ± 0.92
5 wt% Ce	6.34 ± 0.15

**Table 2:** Pore properties of MSGs. (BET: Brunauer-Emmett-Teller; BJH: Barrett-Joyner-Halenda)

Mesopore Structure	Dopant	BET Surface Area (m^2^/g)	BJH Desorption Cumulative Pore Volume (cm^3^/g)	BJH Desorption Average Pore Size Diameter (nm)
**Non-ordered**	—	246.7	0.1686	2.8019
**Non-ordered**	5 wt% Ag	307.8	0.2659	3.1422
**Non-ordered**	5 wt% Cu	167.1	0.1546	3.3767
**Non-ordered**	5 wt% Zn	188.3	0.1460	2.8718
**Non-ordered**	5 wt% Ce	250.5	0.1990	3.2015
**Ordered**	—	411.9	0.6119	4.7230
**Ordered**	5 wt% Ag	528.6	0.8676	5.2439
**Ordered**	5 wt% Cu	328.3	0.4232	4.0138
**Ordered**	5 wt% Zn	380.9	0.5015	4.1182
**Ordered**	5 wt% Ce	438.1	0.6747	4.8182

**Table 3:** Vibrational band assignments and approximate peak band wavenumbers for non-ordered and ordered mesoporous undoped and doped glasses, where ns = not seen.

Mesopore Structure	Dopant	ν_as_ (Si-O-Si) cm^−1^	ν (Si-O NBO) cm^−1^	ν_s_ (Si-O-Si) cm^−1^	B (P-O) cm^−1^	r (Si-O-Si) cm^−1^
**Non-ordered**	—	1047	ns	796	556	442
**Non-ordered**	5 wt% Ag	1045	959	794	551	443
**Non-ordered**	5 wt% Cu	1077	936	799	604/566	456
**Non-ordered**	5 wt% Zn	1071	ns	801	559	445
**Non-ordered**	5 wt% Ce	1050	ns	797	555	448
**Ordered**	—	1032	ns	804	568	441
**Ordered**	5 wt% Ag	1035	ns	797	559	440
**Ordered**	5 wt% Cu	1039	ns	801	557	443
**Ordered**	5 wt% Zn	1034	ns	800	556	440
**Ordered**	5 wt% Ce	1035	ns	800	558	441

**Table 4:** Theoretical and experimental wt% compositions of mesoporous silicate glasses.

Mesopore Structure	Dopant	Theoretical Composition (wt%)				Composition by SQ XRF (wt%)			
		SiO_2_	CaO	P_2_O_5_	Dopant	SiO_2_	CaO	P_2_O_5_	Dopant
**Non-ordered**	—	76	13	11	—	73	14	13	—
**Non-ordered**	5 wt% Ag	71	13	11	5	69	14	13	4
**Non-ordered**	5 wt% Cu	71	13	11	5	68	14	13	5
**Non-ordered**	5 wt% Zn	71	13	11	5	70	13	14	3
**Non-ordered**	5 wt% Ce	71	13	11	5	68	13	14	5
**Ordered**	—	76	13	11	—	76	15	9	—
**Ordered**	5 wt% Ag	71	13	11	5	78	10	10	2
**Ordered**	5 wt% Cu	71	13	11	5	80	9	9	2
**Ordered**	5 wt% Zn	71	13	11	5	72	13	13	2
**Ordered**	5 wt% Ce	71	13	11	5	77	11	8	4

**Table 5:** Theoretical and experimental mol% compositions of mesoporous silicate glasses.

Mesopore Structure	Dopant	Theoretical Composition (mol%)				Composition by SQ XRF (mol%)			
		SiO_2_	CaO	P_2_O_5_	Dopant	SiO_2_	CaO	P_2_O_5_	Dopant
**Non-ordered**	—	80	15	5	—	78	16	6	—
**Non-ordered**	5 wt% Ag	79	15	5	1	76	17	6	1
**Non-ordered**	5 wt% Cu	76	15	5	4	74	16	6	4
**Non-ordered**	5 wt% Zn	76	15	5	4	76	15	6	3
**Non-ordered**	5 wt% Ce	78	15	5	2	76	15	7	2
**Ordered**	—	80	15	5	—	79	17	4	—
**Ordered**	5 wt% Ag	79	15	5	1	83	12	4	1
**Ordered**	5 wt% Cu	76	15	5	4	84	10	4	2
**Ordered**	5 wt% Zn	76	15	5	4	78	15	6	1
**Ordered**	5 wt% Ce	78	15	5	2	82	13	4	1

## Supplementary Materials

The following are available online at https://www.mdpi.com/1996-1944/13/4/992/s1, Figure S1: FTIR spectrum of ordered 5 wt% Cu-MSG, Figure S2: FTIR spectrum of ordered 5 wt% Cu-MSG focused to the specific region of 3000–3700 cm^−1^ wavenumber.
