# Supplementary material for: A Structural Comparison of Ordered and Non-Ordered Ion Doped Silicate Bioactive Glasses
Source: Materials (Basel). 2020 Feb 22;13(4):992. doi: 10.3390/ma13040992 (PMC7078819; doi:10.3390/ma13040992)
Supplement: Supplementary file 1 [file materials-13-00992-s001.pdf]

materials

MDPI

Supplementary Materials

A Structural Comparison of Ordered and Non-Ordered Ion Doped Silicate Bioactive Glasses

Seray Schmitz [1], Ana M. Beltrán [2], Mark Cresswell [3] and Aldo R. Boccaccini [1,*]

[1] Institute of Biomaterials, University of Erlangen-Nuremberg, Cauerstrasse 6, 91058 Erlangen, Germany; seray.kaya@fau.de
[2] Department of Materials and Transport Science Engineering, University of Seville, 41011 Seville, Spain; abeltran3@us.es
[3] Lucideon Ltd., Queens Road, ST4 7LQ Stoke-on-Trent, UK; Mark.Cresswell@lucideon.com
* Correspondence: aldo.boccaccini@ww.uni-erlangen.de; Tel.: +49 (0)9131-85-28601

Received: 21 January 2020; Accepted: 20 February 2020; Published: 22 February 2020

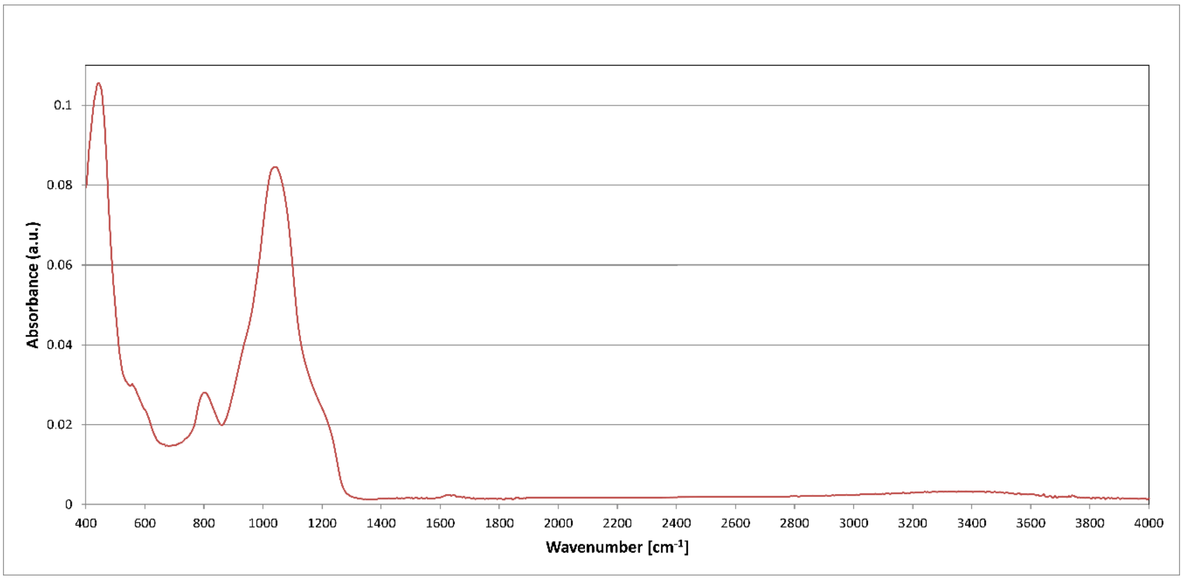

Figure S1. FTIR spectrum of ordered 5 wt% Cu-MSG.

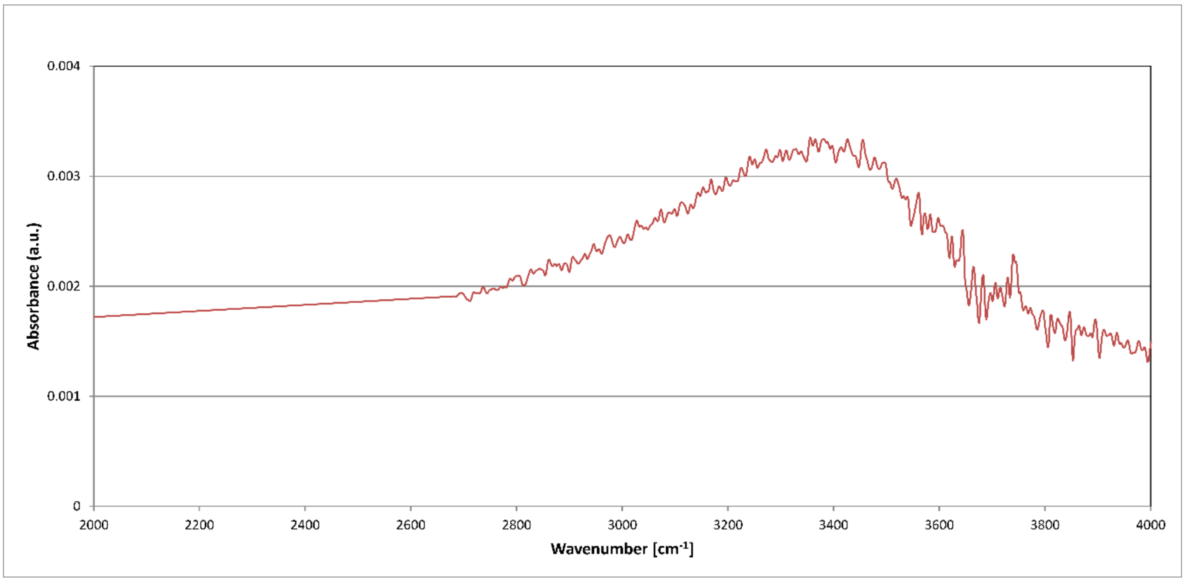

Figure S2. FTIR spectrum of ordered 5 wt% Cu-MSG focused to the specific region of 3000–3700 cm^{-1} wavenumber.
